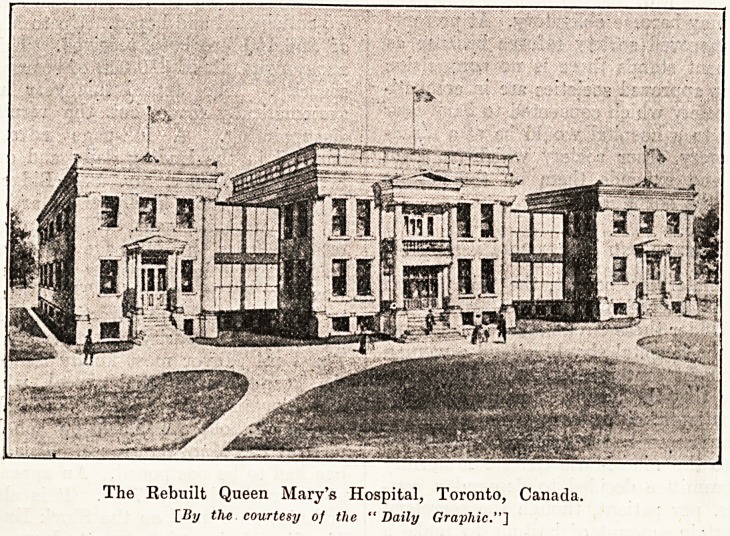# New Canadian Hospital Opened by the Queen

**Published:** 1913-06-14

**Authors:** 


### QUEEN MARY'S HOSPITAL FOR CONSUMPTIVE CHILDREN.

### New Canadian Hospital Opened by the Queen.

THE opening by the Queen of the Hospital for Consumptive Children at Toronto, Canada, last week, by the agency of electricity, from Buckingham Palace is only the latest surprising event in a chequered and interesting history. Opened in 1904, with Mr. W. J. Gage as chairman, under the name of the Free Hospital for Consumptives, with eighty-nine beds, it continued satisfactorily until December 1910, when the greater part of the buildings was destroyed by a disastrous fire, the story of which formed the subject of a paper on "The Prevention of Fire," by Dr. W. J. Dobbie, physician-in-chief to the institution, which he read before the Canadian Hospitals Association, and which subsequently appeared in our columns.

At this point it should be added that three years after the Toronto Free Hospital for Consumptives was opened, a companion institution in the same grounds was also built and received permission to use the title of the King Edward Sanatorium for Consumptives. This latter institution escaped the fire which destroyed the Free Hospital, and the new institution which her Majesty opened last week, and of which the above is a photograph, is the Toronto Free Hospital rebuilt and rechristened by Royal permission the Queen Mary Hospital for Consumptive Children, Toronto.

The story of the institution since its opening in 1904 is thus seen to be full of interest, anxiety and success alternating. Both the hospital and the sanatorium have received striking marks of Royal favour, and the interest in the opening of the rebuilt institution last week by Queen Mary is twofold. In the first place it brings home, thanks to the destruction of distance and natural barriers which it has been the achievement of modern science to secure, that London is the real centre of hospital activity for the whole Empire; and, secondly, there is the interest attaching to the means taken to connect the Queen's room in Buckingham Palace by a special wire with the office of the cable company.

Canadians recall with pride that the foundation-stone of the rebuilt institution was laid by the Duke of Connaught last year, and that it now accommodates a hundred beds. It claims to be the first hospital in the world for consumptive children, and is connected with the National Sanitarium Association of Canada which started seventeen years ago, and of which Mr. Gage, as already mentioned, the first chairman of the hospital, was the founder. Since then two sanatoria have been established in Muskoka and two at Weston. When the first hospital was opened at Muskoka, seventeen years ago, there was no other institution in Canada for the care of consumptives. Now there are twenty-three in the Dominion and fifteen in the Province of Ontario alone, either completed or in the course of construction. When the first hospital was opened at Muskoka fifty patients were cared for; now provision will be found for over five hundred. Altogether nearly six thousand have entered the several homes of the Association. In consequence of the laundries refusing the work of the hospitals through fear of infection a special laundry has been built and equipped at great cost. Another feature of the Association's work is the founding of a school at the Toronto Free Hospital, the first of its kind in a sanatorium.

The Rebuilt Queen Mary's Hospital, Toronto, Canada.
[*By the courtesy of the "Daily Graphic."*]

## Figures and Tables

**Figure f1:**